# Arthroscopic Treatment for Primary Septic Arthritis of the Hip in Adults

**DOI:** 10.1155/2016/8713037

**Published:** 2016-10-05

**Authors:** Jörg Hartmut Schröder, David Krüger, Carsten Perka, Martin Hufeland

**Affiliations:** Center for Musculoskeletal Surgery, Charité-Universitätsmedizin Berlin, Augustenburger Platz 1, 13353 Berlin, Germany

## Abstract

*Purpose.* Primary septic arthritis is a rare differential diagnosis of acute hip pain in adults. Inspired by the success of all-arthroscopic treatment in pediatric patients, we developed a diagnostic and surgical pathway for our adult patients.* Methods.* Seven patients, average age 44 ± 13.7 years with acute hip pain since 4.4 ± 2.9 days in the average, were included. Septic arthritis was confirmed by joint aspiration and dissemination was excluded by MRI and standard radiographs. Surgical treatment consisted of immediate arthroscopic lavage using 4 portals for debridement, high-volume irrigation, partial synovectomy, and drainage.* Results.* Patients were treated in hospital for 12.4 ± 3.1 days (range 7–16 days). WBC and CRP returned to physiological levels. During the mean follow-up of 26.4 ± 19.4 months (range 13–66 months) no patient showed recurrence of infection. The 5 patients with an unimpaired hip joint prior to the infection had a mean modified Harris Hip Score of 94 ± 5.6 points (range 91–100) at final follow-up.* Conclusions.* Arthroscopic therapy using a minimally invasive approach with low perioperative morbidity for the treatment of primary septic arthritis of the adult hip is able to restore normal hip function in acute cases without dissemination of the infection.* Level of Evidence.* IV.

## 1. Introduction

Septic arthritis of the hip is a rare differential diagnosis of acute hip pain in adults [1, 2]. Despite the fact that the hip is the second most affected joint, adult patients with septic arthritis not related to a surgical procedure represent less than 0.00001% of total acute admissions [3–7]. Underlining this a recent multicenter study by Muñoz-Mahamud et al. [8] reviewing the experience in 6 orthopedic departments from 1993 to 2009 could only identify 18 cases.

Symptoms include acute painful hip motion with avoidance of weight bearing but can be variable and unspecific especially in children and elderly, multimorbid patients. For infections not related to injuries or medical intervention the most frequently detected pathogens are* Staphylococcus aureus* and* Streptococcus pyogenes* [6, 9, 10]. In acute septic arthritis plain radiographs may show no bony abnormality, but ultrasound scanning reveals intra-articular effusion. Furthermore, an additional MRI scan can exclude extra-articular abscess formations. Joint fluid aspiration is mandatory and confirms the diagnosis. Synovial fluid white blood cell (WBC) count of >50,000 cells/mm^2^ with a high percentage of polymorphonuclear cells is considered diagnostic for septic arthritis although lower counts have also been observed [11, 12]. Septic arthritis can be present even when the subsequent cultures of blood and synovial fluid are sterile [13].

Complications as a result of nondiagnosed septic/infectious arthritis are chronic osteomyelitis, extra-articular abscess formations, pathologic dislocation, and sepsis. Overall, mortality rates up to 13% have been reported especially in the elderly, multimorbid, or immunosuppressed patients [2, 14]. Joint preserving surgical options, with arthrotomy, lavage, and drainage, are established but may result in prolonged treatment and a higher complication rate [15]. In addition, the sealing of the central compartment by the labrum makes a lavage and debridement of the cartilage area difficult in open surgery without traction. Inspired by the favorable results of arthroscopic treatment for septic arthritis of the knee and the pediatric hip, first reports also for the adult hip have been published [16–19].

However, due to the low incidence with small case series being published, there remains uncertainty about the success of arthroscopic treatment. The purpose of our study was a prospective evaluation of arthroscopic treatment for septic arthritis of the hip in adults. We followed a defined clinical pathway for all patients with ultrasound and MRI examinations on admission, diagnosis confirmation by aspiration, and immediate arthroscopic lavage.

*Hypothesis. *In patients with a contained infection of the hip joint, emergency arthroscopic lavage is able to eradicate the infection and prevent functional impairment.

## 2. Methods

### 2.1. Patients and Diagnostic Path

**Figure 1:**
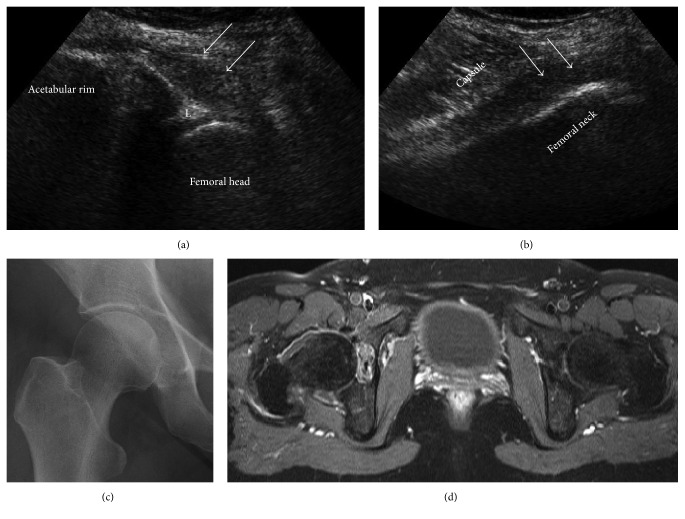
Diagnostic path for patient #4, a 55-year-old woman with a 3-day history of acute pain in the right hip. Ultrasound scans revealed an intra-articular effusion (white arrows; L = labrum) with distension of the capsule (a, b) whereas the radiograph was unremarkable. T2 weighted MRI scans (d) confirmed effusion in the right hip and ruled out extra-articular abscess formation and advanced osteochondral involvement.

13 patients (14 hips, one bilateral infection) were treated for primary septic arthritis between 2007 and 2013 in our institution. Patients with secondary septic arthritis after surgery and implant related infections were not included. Two patients underwent open debridement as the preoperative imaging revealed extra-articular dissemination of the infection. Four patients (5 hips) showed advanced joint destruction due to delayed presentation (22 days in the average) and were treated with Girdlestone's procedure and subsequent total hip arthroplasty. Three female and 4 male patients (Table 1) with an average age of 44 ± 13.7 years (range 26–63 years) were admitted to our emergency department with an acute painful hip since 4.4 ± 2.9 days in the average (range 2–10 days). Standard laboratory tests including blood cultures were conducted which revealed elevated CRP and WBC levels. All patients underwent a diagnostic path prior to surgery with ultrasound scans for joint effusion and biplane radiographs and MRI studies being carried out on the day of admission (Figure 1).

Synovial fluid was analyzed after diagnostic joint aspiration revealing intra-articular pus collection and high white cell counts over 50.000 per cubic millimeter in all patients.

Patient #7, a 42-year-old male patient, had been diagnosed with septic arthritis after he underwent intra-articular injections with hyaluronic acid for initial osteoarthritis. He was first treated solely with antibiotics before being referred to our clinic with rising CRP levels and prolonged pain 10 days after diagnosis. Patient #6, a 44-year-old male, had been diagnosed with a primary perineal sarcoma in 2005 and underwent operative resection and high-dose radiation therapy resulting in a flexion contracture of the hip with markedly remaining soft tissue alterations of the left thigh and recurrent superficial soft tissue infections for whom he was admitted initially. Despite those functional deficits the joint was otherwise unimpaired before being diagnosed with septic arthritis. The other 5 patients' histories were unremarkable with no history of trauma, excessive exercise, or any event that could be taken into account for the onset of the pain. None of the patients was suffering from diabetes and rheumatoid arthritis was on immunosuppressive medication or had received an injection or intravenous medication recently.

### 2.2. Preoperative Imaging

Standard biplane radiographs were unremarkable in 6 of the included patients, showing a well-preserved joint space. The radiographs in patient #7 showed the known initial osteoarthritis for which he had received intra-articular injections as symptomatic treatment.

**Figure 2:**
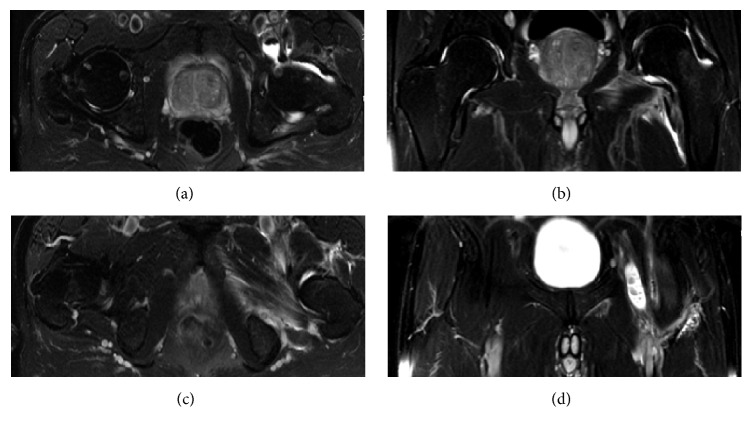
T2 weighted MRI confirms effusion (a, b) with inflammation of the capsule and adjacent muscles and excludes relevant extra-articular abscess formations. Notice the affection of the iliopectineal bursa communicating with the hip joint (a, d).

MRI studies confirmed the intra-articular fluid collection and thickening of the synovia (Figure 2). Osteochondral lesions in the femoral head or the acetabulum, presence of osteomyelitis, and extra-articular disseminations of the infection were excluded in all patients prior to the arthroscopic treatment.

### 2.3. Surgical Technique

Surgical treatment protocol consisted of immediate arthroscopic intervention with the patient placed in supine position as described by Byrd [20] using 4 portals for debridement and partial synovectomy and high-volume irrigation. Debridement and lavage were then first carried out without traction in the peripheral compartment and second in the central compartment. We used a high-volume lavage (minimum 30 liters) with physiological saline. No antibacterial agents were used for irrigation due to possible chondrotoxic influences. In order to prevent fluid leakage and therewith potential spreading of the infection in the surrounding soft tissue, a capsulotomy was not performed. Arthroscopic findings were graded according to the classification for septic arthritis established by Gächter [21] as follows:

 
*Stage I*: opacity of fluid, redness of the synovial membrane, possible petechial bleeding, and no radiological alterations;
 
*Stage II*: severe inflammation, fibrinous deposition, pus, and no radiological alterations;
 
*Stage III*: thickening of the synovial membrane, compartment formation (“sponge-like” arthroscopic view), and no radiological alterations;
 
*Stage IV*: aggressive pannus with infiltration of the cartilage, possibly undermining the cartilage, radiological signs of subchondral osteolysis, and possible osseous erosions and cysts.

After synovectomy and irrigation, two suction drainage systems were inserted in the peripheral compartment. The first drainage was placed in the anterior portal encircling the femoral neck medially; the second was placed in the anterolateral portal to ensure sufficient drainage from the lateral and posterior side. However, continuous postoperative intra-articular irrigation was not conducted. Three patients with an intraoperative stage III according to Gächter underwent a scheduled second-look arthroscopy.

### 2.4. Postoperative Rehabilitation

The suctions drains were removed after surgery depending on the volume of fluid drained. Mobilization from the first day after surgery with partial weight bearing for 3 weeks using crutches was administered. Partial weight bearing was administered to promote soft tissue healing and to reduce pain and protect the vulnerable joint cartilage in the inflammation phase. To prevent capsular fibrosis and adhesions, continuous passive motion was applied after removal of the drains. Intravenous antibiotics followed by oral administration were administered for the duration of minimum 4 weeks postoperatively as recommended by García-Arias et al. and official German guideline on bacterial joint infections [22, 23].

## 3. Results

### 3.1. Intraoperative Findings

**Figure 3:**
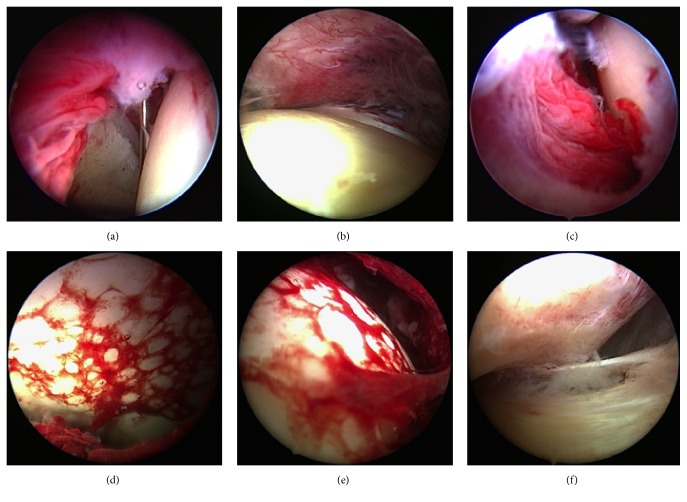
Arthroscopic views revealing severe synovial and capsular inflammation (a–c), fibrinous deposits on the surface of the femoral head (c and e), and pus collection in the capsular compartments (f).

Patients #1, #3, #4, and #5 showed severe inflammation with diffuse synovial injections, various amounts of pus, and fibrinous depositions (Figure 3) but without advanced cartilage alterations (stage II). Patients #2, #6, and #7 were graded Gächter stage III and underwent a scheduled second-look arthroscopy.

### 3.2. Microbiology

Blood cultures obtained from all patients remained sterile. In patients #2 and #3* Staphylococcus aureus* could be identified in the synovial fluid. The gram-stain of the synovial fluid of patient #4 revealed gram-positive cocci, but the subsequent culture remained sterile. In patient #6* Streptococcus agalactiae* and in patient #7* Staphylococcus epidermidis* could be identified. However for patients #1 and #5 the culture remained sterile. In regard to the antibiotic regime Ampicillin/Sulbactam was administered for patients #1 and #5. Patients #2, #3, and #4 were treated with Levofloxacin additionally. Patient #6 received Amoxicillin and patient #7 received Levofloxacin/Rifampicin. The search for an origin of the infection remained negative in all patients except #4 where leukocyte scintigraphy revealed a Dentogen focus and patient #7 who had received intra-articular injections prior to the infection.

### 3.3. Outcome

The patients were discharged from hospital after 12.4 ± 3.1 days (range 7–16 days) in the average. Postoperative recovery was according to expectations in all patients and the pain level was markedly reduced during postoperative mobilization. WBC and CRP levels decreased to physiological levels. During the clinical follow-up of 26.4 ± 19.4 months in the average (range 13–66 months), no patient showed recurrence of the infection. The patients with unimpaired joint prior to the infection (#1–#5) showed full range of motion of the hip joint with a mean modified Harris Hip Score of 94 ± 5.6 points (range 91–100) at final follow-up.

## 4. Discussion

Septic arthritis of the adult hip, not associated with prior surgery, is a rare but serious cause of acute hip pain, which can lead to rapid joint destruction if diagnosis and treatment are delayed [24, 25]. For patients with radiological signs of osteochondral involvement resembling Gächter stage IV, extra-articular dissemination of the infection, or when the option for arthroscopic surgery in the facility is not available, open surgery with arthrotomy remains the treatment of choice. When severe destruction of the hip is already present, joint preserving therapies are seldom successful and Girdlestone's procedure with two-step total hip arthroplasty is indicated [1, 26–28]. In pediatric patients as well as for the knee joint, arthroscopic surgery for septic arthritis is well established and favorable results in regard to joint preservation are reported [29–34]. There are only few studies available which report the results of joint preserving surgery of primary septic arthritis in the adult hip [8, 14]. Muñoz-Mahamud et al. retrospectively analyzed 18 cases (1993–2009; 6 orthopedic centers). Specific data on osteochondral involvement at diagnosis or the timespan from symptoms to surgical intervention was not provided. Here, the first-line treatment consisted in 15 patients of an arthrotomy for debridement and lavage and in 3 patients of resection arthroplasty [8].

Arthroscopy allows access to the hip joint with minimal muscle and especially capsular damage also when a repeated washout is indicated. Direct visual assessment of the articular surfaces in the central compartment under traction and removal of fibrinous deposits are clear advantages in comparison to an arthrotomy. In the only available study comparing arthroscopy versus arthrotomy for the treatment of septic arthritis in children, arthroscopic treatment resulted in a significantly shorter duration of hospitalization and a better clinical outcome but with equivalent infection control [35].

Blitzer and Bould et al. [16, 17] each reported the first cases where arthroscopic management, using a single-portal approach for the hip, was performed.

In the last decade, arthroscopic surgery of the hip has rapidly developed and surgery with the patient in supine position using 3 to 4 portals is now the established standard. In order to prevent iatrogenic injury to the joint cartilage access to the joint should be performed through the peripheral compartment [36]. Kamiński et al. reported treatment by miniarthrotomy through a modified Watson-Jones approach, fenestration of the anterior capsule, and arthroscopic inspection of the joint under traction with good functional results and a sufficient infection control [37].

Limited case series of arthroscopic treatment provide favorable results, but there is still inconsistency in regard to the number of portals used, the insertion of suctions drains, and the necessity for a scheduled second-look arthroscopy [18, 19, 30]. Our patient population is considerably young without relevant comorbidities except patient #6 who underwent resection of a sarcoma and adjuvant radiotherapy years prior to the infection of the hip joint. All were outpatients who complained of a sudden onset of significant hip pain. This constellation might have facilitated the in-time diagnosis, shortened the time to treatment, and made arthroscopic treatment possible. In addition to the standard diagnostics before surgery we prefer an immediate MRI scan in order to exclude associated osteomyelitis or abscess formation ultimately precluding arthroscopic treatment.

We prefer to insert drains for a minimum of two and a maximum of four days to provide sufficient drainage and to have a visual control for the amount and consistency of the joint fluid the first days after surgery. In contrast to that Nusem and Yamamoto do not insert drains due to the risk of secondary contamination and of infection with new bacteria as they proclaim [38]. On the other hand, Kim et al. as well as Kusma et al. in their recent technical note also prefer the insertion of drains [18, 39].

A scheduled second look for further irrigation, direct visual control of the synovia and cartilage condition, and reduction of the risk for intra-articular adhesions was our opinion indicated in three patients (#2, #6, and #7) with advanced infection resembling Gächter stage III. For joints other than the hip, it could be shown that a secondary arthroscopic washout can be beneficial for infection control in patients presenting with Gächter stage II and above in the first surgery [40–42]. In the case series reported by Stutz et al. in Gächter stage III, one course of arthroscopic irrigation and debridement was sufficient to cure the infection in only 3 of the 12 infected joints; a second arthroscopic procedure was necessary in 4, a third in 2, and a fourth in 1 joint [41]. Stutz and Gächter published the results of 46 patients; here all patients with Gächter stage I or II disease experienced full resolution of the infectious process, compared to 78% and 67% of patients with stage III and IV disease, respectively. Of the 9 patients with Gächter stage III, 5 (55.5%) required a second arthroscopy [42]. However, these studies do not focus on the hip joint. We further agree with Kusma et al. that, in stages of an advanced infection above Gächter II, a synovectomy is indicated [39].

These variables in treatment remain to be studied in comparative multicenter studies but taking into consideration that hip arthroscopy is surgically demanding technique with an exceptional steep learning curve sole arthroscopic treatment may remain limited to arthroscopic centers and arthrotomy optionally with arthroscopic assistance as described by Kamiński et al. [37] might be a considerable option for clinics where equipment and expertise for sole arthroscopic treatment are not available. Our study shows that when early diagnosis is achieved, in this considerable large number of patients without extra-articular dissemination of the infection, arthroscopic management is a promising minimally invasive option for the successful treatment of primary septic arthritis in adults with eradication of the infection in all patients and an excellent functional outcome.

In our institution, hip arthroscopy is now the standard of treatment for acute septic arthritis of the adult hip without advanced osteochondral lesions (Gächter stages I–III) or extra-articular abscess formations. In the more advanced stage IV open surgery remains the treatment of choice and usually an unsatisfying outcome is to be expected. To further establish arthroscopic treatment for this indication prospective multicenter studies following a consensus protocol for the diagnostic pathway and comparison to control groups preferring open surgery are necessary.

### 4.1. Limitations

The primary goal of our study was to evaluate infection eradication by arthroscopic lavage in adult patients for which the limited follow-up is sufficient. However, our results do not allow conclusions in regard to the long-term functional and radiographic outcome. The small number of patients in our and other available studies is based on the low incidence of acute primary septic arthritis of the hip.

## 5. Conclusions

Arthroscopic therapy using a minimally invasive approach with a low perioperative morbidity for the treatment of primary septic arthritis of the adult hip is able to restore normal hip function in acute cases without dissemination of the infection.

## Figures and Tables

**Table 1:** Patients' data.

#	Age (y)	Sex	Follow-up (m)	Duration of symptoms (d)	WBC/nl	CRP mg/dl	Culture
1	26	m	38	2	14,9	1,6	No growth
2	63	m	14	2	10,9	31,3	*S. aureus*
3	50	w	16	4	16,2	12,6	*S. aureus*
4	55	w	66	3	12,2	14,9	Gram-positive cocci
5	27	w	13	3	9,8	1,8	No growth
6	44	m	18	7	9,3	15,4	*S. agalactiae*
7	42	m	20	10	7,1	10,0	*S. epidermidis*
